# The Life Mission Theory VII. Theory of Existential (Antonovsky) Coherence: A Theory of Quality of Life, Health, and Ability for Use in Holistic Medicine

**DOI:** 10.1100/tsw.2005.47

**Published:** 2005-05-06

**Authors:** Søren Ventegodt, Trine Flensborg-Madsen, Niels Jørgen Andersen, Joav Merrick

**Affiliations:** ^1^ The Quality of Life Research Center, Teglgåstrdstæde 4-8, DK-1452 Copenhagen K, Denmark; ^2^ The Research Clinic for Holistic Medicine and the Nordic School of Holistic Medicine, Copenhagen, Denmark; ^3^ The Scandinavian Foundation for Holistic Medicine, Sandvika, Norway; ^4^ Norwegian School of Management, Sandvika, Norway; ^5^ National Institute of Child Health and Human Development and Center for Multidisciplinary Research in Aging, Faculty of Health Sciences, Ben Gurion University of the Negev, Beer-Sheva and Office of the Medical Director, Division for Mental Retardation, Ministry of Social Affairs, Jerusalem, Israel

**Keywords:** quality of life, QOL, philosophy, human development, holistic medicine, public health, salutogenesis, sense of coherence, personal development, ego, higher self, coherence, love, joy, consciousness, sex, intent, talent, Antonovsky, Denmark, Norway, Israel

## Abstract

A theoretical framework of existential coherence is presented, explaining how health, quality of life (QOL), and the ability to function were originally created and developed to rehabilitate human life from an existential perspective. The theory is inspired by the work of Aaron Antonovsky and explains our surprising recent empirical findings—that QOL, health, and ability primarily are determined by our consciousness. The theory is a matrix of nine key elements in five layers: (1) coherence; (2) purpose and talent; (3) consciousness, love, and physicality/sexuality; (4) light and joy; and (5) QOL/meaning of life. The layer above causes the layer below, with the layer of QOL again feeding the fundamental layer of coherence. The model holds the person responsible for his or her own degree of reality, happiness, and being present. The model implies that when a person takes responsibility in all nine “dimensions” of life, he or she can improve and develop health, the ability to function, all aspects of QOL, and the meaning of life. The theory of existential coherence integrates a wide range of QOL theories from Jung and Maslow to Frankl and Wilber. It is a nine-ray theory in accordance with Gurjieff's enneagram and the old Indian chakra system. It can be used in the holistic medical clinic and in existential coaching. Love is in the center of the model and rehabilitation of love in its broadest sense is, accordingly, the essence of holistic medicine. To know yourself, your purpose of life (life mission) and talents, and taking these into full use and becoming coherent with life inside and reality outside is what human life is essentially about. The new model has been developed to integrate the existing knowledge in the complex field of holistic medicine. Its strength is that it empowers the holistic physician to treat the patient with even severe diseases and can also be used for existential rehabilitation, holistic psychiatry, and sexology. Its major weakness is that it turns holistic medicine more into an art than into a science because the physician must master intent, which is a poorly understood dimension of existence.

## INTRODUCTION

We have recently concluded our research from 1991–2004 and found that health and the ability to function, in private as well as in our professional life, primarily seem to be determined by our own consciousness[1,2,3,4,5]. This was a surprising finding, as most social and medical researchers have tended to believe that DNA, with other physical and chemical aspects, is what determines the core values of man. The conclusion that consciousness is of major importance has led us to deep reflections on the nature of human life and existence. This does not rule out the important role of a biological mechanism in many diseases, since defect genes and DNA still impact the human being and create problems of health and ability, which often need more mechanical- than consciousness-oriented solutions. On the other hand, most diseases seem to be strongly influenced by both lifestyle and consciousness, i.e., many types of cancer, autoimmune diseases like diabetes, arthritis, and most forms of chronic pain. Most existential, mental, and sexual disturbances also seem connected to psychosocial dimensions like traumatic life events and lifestyle[1,2,3,4,5]. In relation to all these sufferings, we hope that the suggested model will empower the holistic physician by integrating most of the existing knowledge on holistic medicine, personal development, and quality of life (QOL) and making it immediately useful in the clinic.

From an existential perspective, life is a flow (compare the concept of modern physics that everything is [in] a flux of energy and information) and when we are fully alive, we are in flow[6]. Our life is a part of the grand web of life on this planet and we participate through what we take in from the world and what we put out in it, on all levels from matter to consciousness. We perceive, we process, and we find meaning[7] and in this way, we impact the world through our being, our words, and our actions.

The most prominent researcher in the field of the coherence of life was the late medical sociologist Aaron Antonovsky (1923–1994) from Ben Gurion University of the Negev in Israel, who claimed that the sense of coherence was the most fundamental quality in the good, healthy, and able life created by our understanding, leading to our actions, leading to our meaning of life, and leading us to new understanding (see Fig. 1)[8,9].

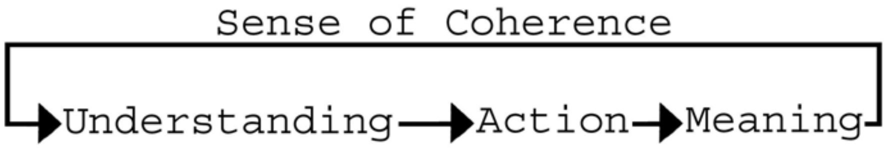

**FIGURE 1**. Sense of coherence created by Antonovsky was based on understanding, power of action (manageability), and meaningfulness (simplified presentation, see [8,9] for details).

We believe that the conceptualization of coherence made by Antonovsky was in some way a “male-oriented”, mentally focused concept and perceive that one of the major weaknesses was the lack of focus on emotions and the modest weight the model placed on love and sexuality. We have recently found that answers to questions related to feelings statistically showed a stronger connection to self-evaluated physical health than answers to questions related to understanding and action (work in progress). A more "female" and bodily-emotionally-spiritually focused concept (see Fig. 2) was presented by Henry Kimsey-House[10] using a special understanding of the concept of responsibility, arising from *feeling and noticing the urge of self and the world*, allowing oneself *to react on such an urge — in oneself, in the space, or in the world at large — with full permission*, and always *noticing the impact on self, others, and the world*. As an event develops, a new urge begins to build and, with permission from the self, leads to new actions and impact. The concept of full permission to move on the urge is beautiful as it takes us right into the flow of life, responding to everything that comes into our consciousness, not straining us in the action, but simply going with the flow of the moment, like the completely relaxed supreme performer of the martial arts or the Japanese Zen bow master perfectly hitting the center with no effort at all.

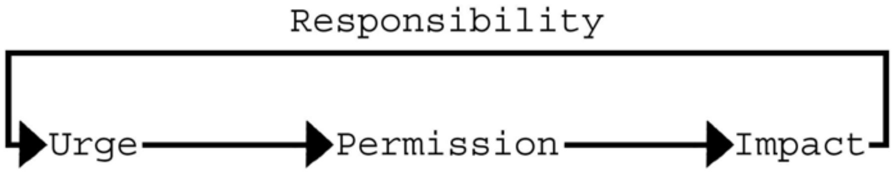

**FIGURE 2**. Coherence through the female concepts of urge felt within self or in the outer world, full permission to act on the urge, and letting the resulting impact be a part of the next moment's urge[26].

Another way of putting it would be that in the optimal, coherent, and healthy life, we notice all the gaps in our reality, all the things that are not exactly the way we want them to be, and respond to them. Philosophically explained, the reason that we perceive these gaps is our fundamental purpose of life, organizing our consciousness and making us want to close any such gap we perceive (mentally as understanding; bodily, emotionally, and spiritually as an urge), which is our essential job in life[11,12,13,14,15,16,17,18,19,20,21,22,23,24,25].

The concepts of understanding and urge can be joined in the concept of health on all levels of existence (physical, emotional, mental, and spiritual), the concepts of action and permission can be joined in the concept of function, and the concepts of meaning and impact can be joined in the concepts of QOL (see Fig. 3).

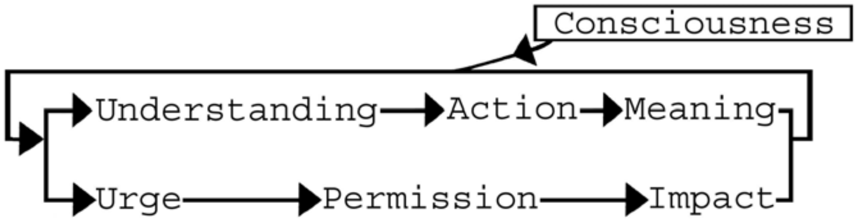

**FIGURE 3**. Abstracting the male and female concepts of existence in coherent flow into the three core values of the human being: health, ability, and quality of life (QOL).

The theoretical challenge is how to take the essence of human existence (both the male and female part) in a coherent flow into a theory. We have called our model *existential coherence*, or *Antonovsky coherence*, to honor Aaron Antonovsky for originally presenting the brilliant idea of coherence and thus pioneering the field. The challenge is to model existential coherence in such a way that all the relevant aspects of human life and existence are included, so that the theory can guide the process of development of flow and optimal life.

Please allow us to suggest such a theoretical framework for the existential coherence, which centers on the concept of love, which includes both mental and physical aspects of life, but also contains the duality of understanding and urge (consciousness and sex). It is developed for use in the holistic medical clinic, in coaching and development of business companies and organizations, their leaders and employees, with the hope that it will also be used for training in medical schools and educational institutions, where there is the intent to develop, recover, or rehabilitate human existence in all aspects from QOL, health, to the ability to function, privately as well as professionally.

## A THEORECTIAL FRAMEWORK FOR EXISTENTIAL (ANTONOVSKY) COHERENCE

The theory of existential coherence (see Fig. 4) states that QOL, our experience of meaning, truth, good, beauty, value, good relationships, fulfillment, etc. in life is caused by our ability to function physically, emotionally, mentally, spiritually, and sexually. Our ability to function is in our thinking, caused by our physical, mental, spiritual, sexual, and social health, which allows us to express our purpose of life and use all our physical, emotional, mental, spiritual, sexual, and social talents. *Meaning* and the other dimensions of *global QOL* seem to us to be born out of two subjective, highly mysterious dimensions that we call *light* and *joy*. Light is a subjective quality of *consciousness and love*, while joy is a subjective quality of *love and physicality*, or *love and sexuality*, if sex is taken to be the fundamental force moving the subjective life energy. The fusion of light and joy into *meaning* is one of the most profound mysteries of the conscious existence; but as mysterious as it is to our minds, which seek mechanistic explanation, it is as self-evident to our intuition (see Fig. 4).

As modern physics explains to us, the world is a web of energy and information. The living organism seems to be an independent creature with a purpose and life of its own; but this autonomy is highly illusory, as reality has many levels of depths, and everything in the end is part of the *web of reality* and every purpose of life is related to the surrounding world and born out of this relationship. Gautama Buddha (566–486 BCE), the founder of Buddhism, thought that everything is relative and, in a state of deep wisdom, we realize that even what we call the soul, the core of our independent existence, is illusory and everybody is existentially rooted in the creative emptiness of the universe, sunyata. Jesus Christ, the founder of Christianity, thought similarly that we are all children of God[27]; when we come to know our true nature, we come to know God. It seems essentially to be the core lesson of all the religions of the world from the Sufi mysticism to the Jewish Kaballah and the Native Americans. We are, in the most fundamental existential aspect, one with the universe. We never were separated because we cannot get out of this world that has born us. The universe is our true womb and the umbilical cord can never be cut. Out of this web of energy and information, out of this world, comes *intent* as we see it, which gives birth to the mind, *consciousness*, and *talent*, which basically is informational “templates” or “jigs”, which give birth to the *body, its organs, and sexuality*. The *coherence of the world* is thus the causal dimension in the theory of existential coherence (see Fig. 4).

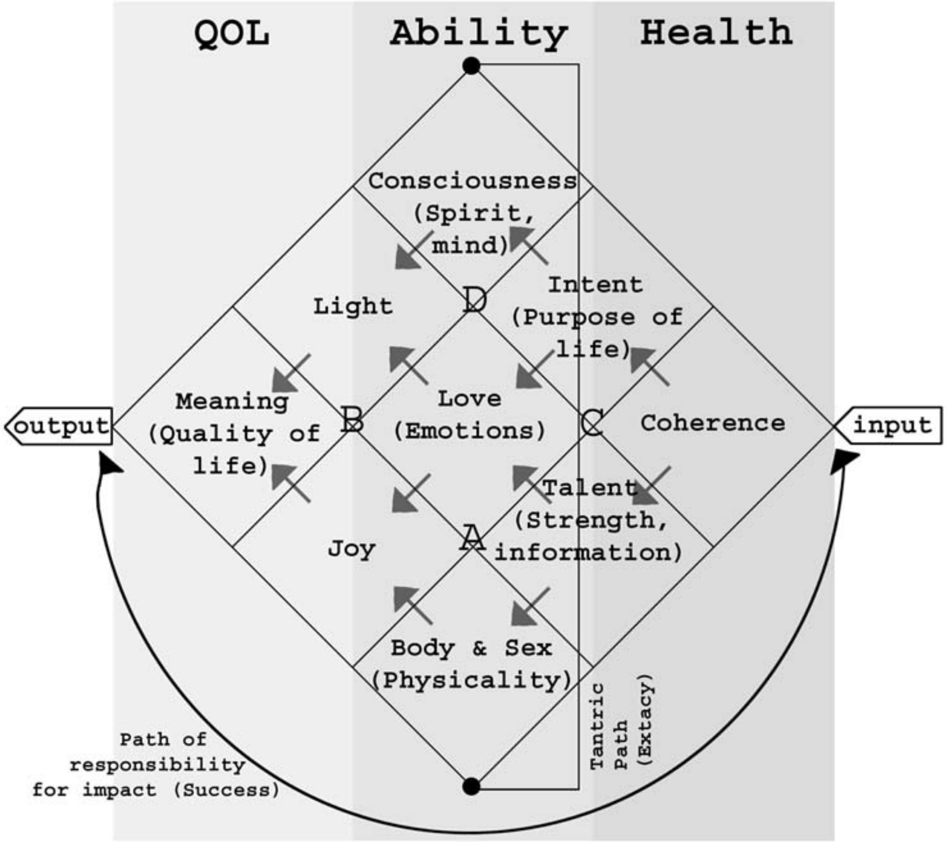

**FIGURE 4**. The theory of existential (Antonovsky) coherence. The framework contains nine key concepts in five layers: (1) coherence; (2) intent/purpose, talent/strength; (3) consciousness/mind, love/emotions, physicality/sexuality; (4) light, joy; (5) QOL/meaning. The layer above causes the layer below, with the layer of QOL again feeding the layer coherence. The model thus holds the person responsible for his or her own degree of reality, health, happiness, and success in life. (Note that the four squares centered by the letters A, B, C, and D to some extent correspond to the old alchemic, metaphysical elements of earth (solid, body), water (fluent, emotions), air (spirit and mind), and fire (transformation, creation). The path of responsibility is noticing and reacting to our own impact on the world. The path of tantra is integrating consciousness, love, and sexuality.

Intent is really a mysterious power; it gives birth to our purpose of life (see life mission theory[12]), it activates all our talents[14], and it is also father to the evil in us[16]. Nobody seems to be able to explain the true nature of intent; it is a direct creative force that manifests itself without noticeable steps. Cultivation of the use of intent is the secret of sorcery[28] and mobilization of talent with intent is, as we see it, the secret of holistic medicine[29,30,31,32,33,34,35,36,37,38,39,40,41,42,43,44,45,46,47,48,49,50, 51,52,53,54,55,56,57,58,59,60]. We use intent without knowing how. We intend it. The path of understanding ends blindly. This is what gives the life mission theory its strange feeling; the purpose of life just emerges out of the coherence of the world. To understand this, imagine yourself as the first cell, the zygote, orienting yourself, looking for the most valuable stake in this life, choosing a purpose and developing your consciousness, personality, character, understanding, wishes, and behavior from that. Such an idea of an "early contract with the spirit" is often found in the medical systems of premodern cultures. White Eagle, a Native American healer, wrote to us about the path of a patient healing her cancer: "…she must find the real reason she came to this lifetime, find her gift to give and remember her original agreement with the Great Spirit for the purpose of the gift of this lifetime. When she remembers this, then she must choose to give herself fully to her agreement, no matter what the pathway is. Then, she will live."

Talent is no less mysterious. Modern molecular biology states that all the information of the organism is contained in the chemical composition of the DNA molecule, but recent research (discussed in[18,19,20,21,22,23,24,25]) has demonstrated that this hypothesis is presumably wrong. The form of the body, the structure of the brain, the distribution of the cells in our body, our memory, and general generic and constructive input from the world is not from the genes, but from a much more complex and hidden source, which is an inherent part of the web of the world. So all our talents seem to be sourced by the same dark and invisible side of the universe as intent.

Out of the combined action of talent and intent arise all the beautiful structures of our biology, our body, our mind and brain, our sexuality, our purpose of life, and character of our personality, but most importantly our love for life and our love for others and ourselves. This is, to us, the most profound mystery of all, the birth of our love, the birth of our liking, our lust, and our interest in the broad sense of the word. Our interest creates our consciousness, our lust creates our sexuality, but our love creates our life. Our love is born out of our purpose of life; we are here to contribute with everything we got as gifted human beings. We want to contribute by using our unique skills and talents for mankind to make the world a better place and close all the gaps we perceive. The creation of a *me* and a *you*[61], the most brilliant and amazing of illusions, is what makes the world go round. Not money, not physical values, but the need of being of value to you, my dear, my friend, my boss, my beloved, my client, my patient. Our true nature is to be of service in love, living our love, being centered in love.

So the good health, high quality, highly valuable life with self-confidence, self-esteem, and happiness are from an existential point of view created when:

- We are deeply rooted in reality (that is, we are real) and a coherent part of the web of the world.
- We know our purpose of life and master our intent.
- We acknowledge our talents and take them into full use.
- We allow ourselves to be creatures of love, honoring our love for life and the world, ourselves, and others in everything we do and are.
- We use our consciousness to understand all aspects of life.
- We give ourselves full permission to notice and follow the urge we feel within ourselves, in the group (in the space), and in the world.
- We take responsibility for filling our life and our world with light.
- We take responsibility for filling our life and our world with joy.
- We take responsibility for closing all the gaps we perceive and create the meaningful and rich life we deeply want. Happiness comes from living our purpose of life and using all our talents to give what we have to offer with unconditional love.

## PERSONAL DEVELOPMENT AND EXISTENTIAL HEALING

The nine key dimensions of existence are present in a passive and an active form, corresponding to the being and doing of life (see Table 1).

Interestingly, as a person develops, the nine areas merge completely; every part of existence becomes conscious, filled with love, meaningful, joyful, enlightened, purposeful, urge-driven, ecstatic, and coherent, as all parts of existence expand into the neighbor areas. This expansion of all existential areas is the project of personal development; such as sex expands into the consciousness and love expands into sexuality, so do we have the classical art of sexual tantra (see “the path of tantra” Fig. 4, that is integrating sex and consciousness). One by one all the splits and participations that torment modern man heal in this process of existential integration. Existential healing is, therefore, the primary goal of personal development[15,62,63].

**TABLE 1**
**The Nine Key Dimensions of Existence**

| Dimensions | Active Form | Passive Form |
|---|---|---|
| 1. Coherence, the web, the nest of the world | Receiving, taking in | Being an integrated part |
| 2. Intent/purpose of life | Intention, decision | Having a purpose (of life) |
| 3. Talent/strength | Using skills and urges | Having strength and structure |
| 4. Consciousness | Noticing, knowing, understanding, planning | Being awake |
| 5. Love | Acting in love | Being in love |
| 6. Sex/physicality | Meeting, enjoying | Being man/woman of character |
| 7. Light | Bringing light | Being in light/enlightened |
| 8. Joy | Bringing joy | Being in joy |
| 9. Meaning/QOL | Creating/fulfilling life, giving | Being alive, having impact |

## The Relationship Between the Theory of Existential Coherence and Other Related Theories

The theory of existential coherence explains many of the same facets of existence covered by the Four Quadrant Theory of Ken Wilber[62]. He also started with "The Great Nest of Being", what we call the coherent matrix of energy and information, or the web of the world. Wilber's four quadrants are intentions, behavior, culture, and social relations, but love is rejected as a central concept in Wilber's model, making this model less useful for deep holistic, existential therapy, where love, trust, and holding are prerequisites for taking the patient into the state of consciousness we call "being in the process of existential healing"[63,64]. Responsibility for the person's own world is also difficult to rehabilitate using the Wilber model, whereas this is the consequence of walking the path of responsibility, noticing and reacting to your own impacts (see Fig. 4).

An interesting question is how the theory of existential coherence relates to other holistic QOL and health theories. The QOL theories[64,65,66] seem to be contained in the present theory, as the integrated QOL theory[64] has the meaning-love-coherence axis as its core, but only as a mysterious center of existence. Maslow's QOL theory[66,67] (Abraham Maslow, 1908–1970) has transcendence of the ego as its primary perspective with the understanding of the ego presented in the theory of the ego[13], where the ego is the personality organized around a subtalent and transcending the ego means finding your real purpose of life and living through that. This theory is also included in the theory of existential coherence. QOL as realizing the biological potentials is also included, as these potentials are found in both intent and talents. Interestingly, this theory only contains the expression of life, not the impression that the coherence theory contains. A new feature is the parallel focus on consciousness and sexuality, giving sex a much more prominent emphasis in life that the earlier theories. Frankl's theoretical construction[7] (Viktor Frankl, 1905–1997) stressed mostly the meaning of life and did not stress sexuality either, while Jung[69] (Carl Jung, 1875–1961) and especially Sigmund Freud (1856–1939)[70] had most of the focus on sexuality as the driving force of life. This is actually still the case in the present theory as the dimension of sex contains the body and the basic physical energy of life. Later thinkers focused more on love[71,72,73]. Jung included most of the nine dimensions in his work, but did not integrate them into one theoretical framework. His archetype was an exploration of the hidden structures of human talent and his work on nonlocality has been an important contribution to our present-day understanding of the deep and mysterious nature of the phenomenon of coherence. In the present theory of existential coherence, body and sexuality are as important as mind and consciousness for our QOL, health, and ability.

The theory of existential coherence is a nine-ray theory (compare it to the seven-ray theory of human character[17]), similar to the Gurjieff enneagram model (George Ivanovitch Gurjieff, 1972–1949) that maps all purposes of life into nine main categories (see Table 2)[74]. The two frameworks both hold love as the core concept. The enneagram can be seen as the "explosion" of the nine rays[17] into the domain of purpose of life (the active side of the intent/purpose area). It seems also to be somewhat compatible with the ancient Indian chakra system (Table 2).

**TABLE 2**
**Correlation Between the Theory of Existential Coherence and Gurjieff's Enneagram Model (Also Including a Tentative Correlation to the Ancient Chakra System)**

| Coherence Theory Key Concept | Enneagram Key Concept | Chakra System |
|---|---|---|
| 1. Love/emotions | Love | Hearth |
| 2. Light | Perfection | Solar plexus |
| 3. Talent/strength | Will/strength | Throat |
| 4. Coherence | Laws of the universe | Divine energy |
| 5. Sex/physicality | Source/creativity | Root/Hara |
| 6. Meaning/QOL | Omniscience | The whole (nadis) |
| 7. Joy | Trust | Hara |
| 8. Intent/purpose | Divine plan | Third Eye (brain center) |
| 9. Consciousness/mind | Truth | Crown |

## DISCUSSION

The value of any theory is its practical or empirical use, because just giving a perspective or mapping an area of the universe, as in this case the inner landscape of human existence, has no value per se. The proposed theory states that coherence gives birth to intent and talent, which creates health, which creates ability, and using these abilities creates meaning and QOL. If this theory can support the patient's development and recovery of the three important dimensions of QOL, health, and ability, the theory has fulfilled its purpose.

The proposed model seems to be well supported by many poorly understood, but surprising, empirical findings; improvement of QOL has thus caused improved health and survival both for patients with cancer[75] and coronary heart disease[76].

So how can it be used? The primary strength of the theory of existential coherence is that it can be used for problem solving and subsequent holistic healing of QOL, health (mental, emotional, physical, sexual, spiritual), and ability in general. If QOL is poor, there is a lack of light or joy or both, which can be developed through development of consciousness, love, and sexuality with physical, emotional, and bodily presence. Discovering your purpose of life and talents by taking them into use can develop consciousness, love, and sex. Purpose of life and talents can be found by getting real or, in other words, seeking and finding meaning and coherence in life and the deep web of the world. Getting real is really what holistic therapy and personal development is about. In the language of the Native American sorcerer tradition, people are transformed from ghost to real humans by confronting reality in all its aspects and reconnecting to self and others. A patient who is seriously ill and without joy in life and with no close friends is often in a disconnected, unreal state of existence and rehabilitating existence starts basically with plugging the person into reality.

Balancing life is another useful way of using the theory of existential coherence. In coaching, the nine areas can be explored and weakly developed areas can be stressed and highlighted. Exercises can be designed to rehabilitate each of the nine areas (see examples in the many case stories in the series on clinical holistic medicine[29,30,31,32,33,34,35,36,37,38,39,40,41,42,43,44,45,46,47,48,49,50,51,52,53, 54,55,56,57,58,59,60]. Right-left balance means balance between the inner and the outer world. Top-down balance means balance between head/mind and body/sexuality. A good life is centered on love. Bringing love into focus in the patient's or client's life is of utmost importance and the model explains why in a simple and convincing way.

Taking leadership in private and professional life is another important aspect of the model. You succeed as a human being when you assume responsibility for your own life, which according to this model means that you know yourself, your purpose of life and talents, and you fight all barriers and resistance, inner and outer, to bring these gifts to the world and the people dear to you. Existential leadership means that you live your life from your heart and that giving is more important than receiving. In your existential leadership, you bring love, light, joy, meaning, and value to your life and your work, dedicated to develop your consciousness, body, and sexuality still further, to be more real, more skillful, and more daring and direct in your intentions, character, and behavior.

Our new model of coherence has been developed to integrate the existing knowledge in the complex field of holistic medicine. The strength is that it empowers the holistic physician to treat patients with even metastatic cancer and other chronic and severe diseases[75,76]. It can also be used for existential rehabilitation in holistic psychiatry and sexology. Its major weakness is that it turns holistic medicine more into an art than a science, because the physician must master intent, which is a poorly understood dimension of existence.

## CONCLUSION

A theoretical framework of existential (Antonovsky) coherence for use in the holistic medical clinic is presented. It explains how health, quality of life (QOL), and the ability to function were originally created, and developed to rehabilitate human life from an existential perspective. The theory integrates a remarkable number of aspects of earlier scientific, semi-scientific, and religious models of existence and explains our recent surprising empirical findings, namely that QOL, health, and ability primarily are determined by our consciousness[1]. According to the flow of the presented scheme, high QOL is based on good physical, emotional, mental, spiritual, and sexual functioning, which are based on good existential health, by which we mean living your purpose of life and using all your gifts and talents. As the life we create impacts our health, the model is really circular. It is this circular nature of life that explains our own vast responsibility for QOL, health, and ability.

The theory is illustrated by a matrix of nine key elements organized in five layers: (1) coherence; (2) intent/purpose of life, talent/strength/information; (3) consciousness/spirit/mind, love/emotions, body/physicality/sexuality; (4) light, joy; and (5) QOL/meaning. The layer above causes the layer below, with the layer of QOL again feeding the layer of coherence. The model holds the person responsible for his or her own degree of reality, happiness, and being present. The model implies that a person, by taking responsibility for his or her existence in the nine dimensions, can radically improve and develop health, ability to function, and all aspects of QOL and meaning in life.

The theory of existential coherence can be used in the holistic clinic and in coaching, for problem solving, to help balance your patient's or client's life, and to encourage and support your patient/client into taking existential leadership. It is especially useful when the patient needs to take a step deeper into existence, to assume responsibility for his or her own life and thus improve QOL in general and heal from a chronic condition. Love is in the center of the model and rehabilitation of love is, accordingly, the essence of holistic medicine, company and life coaching, and all other kinds of work with the intention of facilitating personal development. To know yourself and your purpose of life and talents, by taking them into full use, becoming coherent with life inside and reality outside, is what life really is about.

The presented model of coherence is best used as a roadmap to existence, allowing the holistic physician to help the patient mobilize his or her inner hidden resources by letting him or her understand the inner logic of the creation of the good coherent life, and helping the patient to assume responsibility step by step for all the nine core areas of life embraced by the model.

## BIOSKETCHES

**Søren Ventegodt, MD,** is the director of the Quality of Life Research Center in Copenhagen, Denmark. He is also responsible for a Research Clinic for Holistic Medicine in Copenhagen and is a popular speaker throughout Scandinavia. He has published numerous scientific or popular articles and a number of books on holistic medicine, quality of life, and quality of working life. His most important scientific contributions are the comprehensive SEQOL questionnaire, the very short QoL5 questionnaire, the integrated QOL theory, the holistic process theory, the life mission theory, and the ongoing Danish Quality of Life Research Survey, 1991–94 in cooperation with the University Hospital of Copenhagen and the late professor of pediatrics, Bengt Zachau-Christiansen, MD, PhD. E-mail: ventegodt@livskvalitet.org. Website: http://www.livskvalitet.org

**Trine Flensborg-Madsen**, **BA, Cand Scient San Publ**., is a research assistant at the Quality of Life Research Center in Copenhagen, Denmark. At this time, she is finishing her masters degree in Public Health at the Department of Public Health, University of Copenhagen. E-mail: tfm@livskvalitet.org

**Niels Jørgen Andersen, MSc,** professor, Department of Innovation and Economic Organization, Norwegian School of Management. This department conducts research and provides teaching in central topics related to innovation, business development, management of global companies, business history, and economic organization. Mr. Andersen's research activities within the Department are related to four core subjects within the discipline: business history, cooperative organizations, business development and entrepreneurship, and finally, studies of industries with a special focus on the electricity industry. He is also the dynamic chairman of the nonprofit organization, Stiftelsen Holistisk Medisin Scandinavia, which aims to support the scientific development, research, and documentation of complementary and holistic medicine in Scandinavia. E-mail: niels.j.andersen@bi.no. Website: http://www.bi.no/users/fgl93013

**Joav Merrick, MD, DMSc,** is professor of child health and human development affiliated with the Zusman Child Development Center, Division of Pediatrics and Community Health at the Ben Gurion University, Beer-Sheva, Israel; the medical director of the Division for Mental Retardation, Ministry of Social Affairs, Jerusalem; and founder and the director of the National Institute of Child Health and Human Development. He has numerous publications in the field of child health and human development, rehabilitation, intellectual disability, disability, health, welfare, abuse, advocacy, quality of life, and prevention. Dr. Merrick received the Peter Sabroe Child Award for outstanding work on behalf of Danish Children in 1985 and the International LEGO-Prize ("The Children's Nobel Prize") for an extraordinary contribution towards improvement in child welfare and well being in 1987. E-mail: jmerrick@internet-zahav.net. Website: www.nichd-israel.com
